# Hopefulness predicts resilience after hereditary colorectal cancer genetic testing: a prospective outcome trajectories study

**DOI:** 10.1186/1471-2407-10-279

**Published:** 2010-06-11

**Authors:** Samuel MY Ho, Judy WC Ho, George A Bonanno, Annie TW Chu, Emily MS Chan

**Affiliations:** 1 Department of Psychology, The University of Hong Kong, Pokfulam, Hong Kong; 2 Department of Surgery, The University of Hong Kong, Queen Mary Hospital, Pokfulam, Hong Kong; 3 Department of Counseling and Clinical Psychology, Teachers College, Columbia University, 525, West 120th Street, Box 218, New York, NY 10027, USA; 4 Hereditary Gastrointestinal Cancer Registry, c/o Department of Surgery, Queen Mary Hospital, Pokfulam, Hong Kong; 5 Hereditary Gastrointestinal Cancer Registry, c/o Department of Surgery, Queen Mary Hospital, Pokfulam, Hong Kong

## Abstract

**Background -:**

Genetic testing for hereditary colorectal cancer (HCRC) had significant psychological consequences for test recipients. This prospective longitudinal study investigated the factors that predict psychological resilience in adults undergoing genetic testing for HCRC.

**Methods -:**

A longitudinal study was carried out from April 2003 to August 2006 on Hong Kong Chinese HCRC family members who were recruited and offered genetic testing by the Hereditary Gastrointestinal Cancer Registry to determine psychological outcomes after genetic testing. Self-completed questionnaires were administered immediately before (pre-disclosure baseline) and 2 weeks, 4 months and 1 year after result disclosure. Using validated psychological inventories, the cognitive style of hope was measured at baseline, and the psychological distress of depression and anxiety was measured at all time points.

**Results -:**

Of the 76 participating subjects, 71 individuals (43 men and 28 women; mean age 38.9 ± 9.2 years) from nine FAP and 24 HNPCC families completed the study, including 39 mutated gene carriers. Four patterns of outcome trajectories were created using established norms for the specified outcome measures of depression and anxiety. These included chronic dysfunction (13% and 8.7%), recovery (0% and 4.3%), delayed dysfunction (13% and 15.9%) and resilience (76.8% and 66.7%). Two logistic regression analyses were conducted using hope at baseline to predict resilience, with depression and anxiety employed as outcome indicators. Because of the small number of participants, the chronic dysfunction and delayed dysfunction groups were combined into a non-resilient group for comparison with the resilient group in all subsequent analysis. Because of low frequencies, participants exhibiting a recovery trajectory (n = 3 for anxiety and n = 0 for depression) were excluded from further analysis. Both regression equations were significant. Baseline hope was a significant predictor of a resilience outcome trajectory for depression (*B *= -0.24, *p *< 0.01 for depression); and anxiety (*B *= -0.11, *p *= 0.05 for anxiety).

**Conclusions -:**

The current findings suggest that hopefulness may predict resilience after HCRC genetic testing in Hong Kong Chinese. Interventions to increase the level of hope may be beneficial to the psychological adjustment of CRC genetic testing recipients.

## Background

Although predictive genetic testing undertaken to identify mutated gene carriers for continued medical surveillance is now possible [1-4], this procedure has important psychological consequences. In a prior study, up to 43% of adults who tested positive for familial adenomatous polyposis (FAP) were clinically anxious after receiving their genetic testing result [5]. In another study on recipients of BRCA1/2 or HNPCC genetic susceptibility testing, 29.3% and 14.1% of participants showed an increase in hereditary cancer-related distress levels at two weeks and six months after test result disclosure, respectively [6]. Other studies, in contrast, have reported that individuals undergoing genetic testing did not experience adverse psychological consequences [7]. Despite these inconsistent findings, some studies have shown that carriers tend to exhibit at least a transient increase in their anxiety levels after disclosure [8]. Psychological distress among mutation carriers is understandable because they have to face the uncertainty of the onset of cancer, the possibility of passing the faulty gene on to their children, and the potential for genetic discrimination [9-15]. Moreover, test-related distress may have a negative effect on compliance with health-protective behaviours [13]. Ho et al provide indirect support for this proposition in a study of 62 hereditary colorectal cancer (HCRC) genetic testing recipients [10]. The researchers found that subjects with higher depression level tended to focus more on the negative consequences of learning their genetic testing results and hence may choose to decline genetic testing. There is a need to identify the factors that affect resilience to HCRC genetic testing so that appropriate intervention can be provided to increase compliance and improve the psychological well-being of those tested.

### Hope and Coping with Cancer

Personal characteristics may have an important effect on adjustment to HCRC genetic testing. Michie et al. [5] concluded from a longitudinal prospective study that HCRC genetic testing subjects who were low in optimism and self-esteem were more likely to be clinically anxious in the first year after testing. The cognitive theory of hope proposed by Snyder and his colleagues [16,17] in helping people to cope with stressors has recently been the focus of much attention. According to this model, hope has three interrelated cognitive components: goals, agency and pathways. Agency refers to an individual's motivation to meet desired goals, while pathways refer to an individual's ability to produce routes to attain these goals [18]. A guiding assumption of Snyder's hope model is that human actions are goal-directed [16] and goals themselves are the cognitive anchors of hopeful thinking [19]. Goals may vary in terms of their time frame (short- or long-term), yet they have to be of sufficient value to the individual to occupy conscious thought [20]. Goals typically contain some degree of uncertainty, yet they must be attainable [21]. Snyder's model proposes that when confronted with negative events such as a positive genetic testing result for HCRC, high-hope individuals will be distressed only temporarily and will bounce back full of energy and ideas on how to achieve their life goals [22]. Research shows that hope is a significant predictor of psychological well-being not only among healthy people [23] but also among individuals with chronic illnesses such as spinal cord injuries [24] and cancer [25-28]. For example, Stanton and colleagues [27] investigated hope and coping strategies as predictors of adjustment among 85 women one year after a diagnosis of breast cancer and reported that high-hope women adopting problem-focused coping strategies adjusted better.

### Prototypical Psychological Outcome Trajectories after a Stressful Event

Although HCRC genetic testing is a stressful event for most people, there are marked differences in how individuals respond to it [29]. Bonanno and his colleagues [30-33] identified four prototypical patterns or trajectory outcomes that capture most people's long-term psychological responses after a potentially traumatic event. The four trajectories are resilience, chronic dysfunction, recovery, and delayed reaction [33].

**Figure 1:**
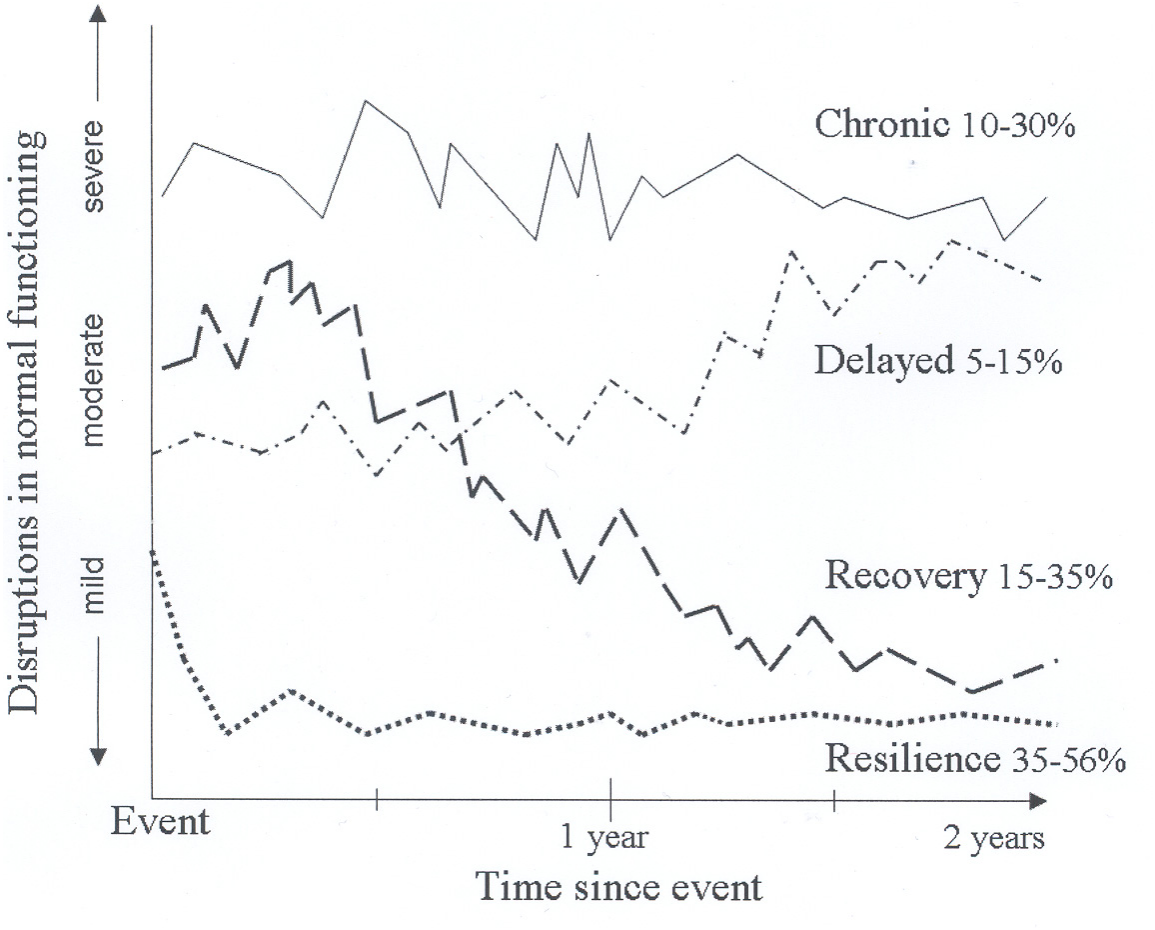
Hypothesized Prototypical Outcome Trajectories after a Stressful Event

*Resilience *is conceptualized as an individual's ability to maintain a relatively stable and healthy level of psychological and physical functioning after a traumatic event [31-35]. People in the *chronic dysfunction *category show a consistent and persistent pattern of elevated symptoms and distress. Typically, only a small percentage of individuals (from 5% to 10%) exhibit this trajectory pattern, but the percentage may vary according to the severity and type of trauma [30]. Another prototypical trajectory outcome, termed *recovery*, represents individuals who initially experience elevations in symptoms and distress, followed by a gradual reduction and return to the population norm. Finally, the *delayed reaction *category includes individuals who initially show moderate (sub-threshold) symptom levels after a potentially traumatic event, followed by a gradual increase to above-threshold elevations over time. These four prototypical outcome trajectories and the estimated proportion of people in each category as observed in previous studies are shown in Figure 1. It should be noted that the percentages of people in each category tends to vary depending on the severity and type of trauma [33,36,37]. For example, Bonanno et al. [30] examined the prototypical outcome trajectories of Hong Kong patients who had recovered from severe acute respiratory syndrome (SARS) and reported a higher percentage of people in the chronic dysfunction category (42%) than in other previous studies.

Most previous studies have used a normative comparison approach that defines health and dysfunction on the basis of established norms for the specified outcome measure to define the outcome trajectories [34,38]. Prototypical trajectories defined in this way are represented graphically in Figure 1. The latent class growth curve model has recently been used to classify people into these outcome trajectories [30] although results similar to those obtained under the normative comparison approach were obtained.

### The Present Study

The prospective longitudinal study reported in this paper was aimed at investigating whether the cognitive style of hope can predict prototypical psychological outcome trajectories after HCRC genetic testing. In particular, we hypothesized that high-hope HCRC genetic testing recipients would have a higher tendency to show a resilience outcome trajectory pattern than their low-hope counterparts. The current investigation makes several contributions to existing research. First, as the only study to have applied the cognitive style of hope to HCRC genetic testing, the findings could have important implications for the psychosocial care of individuals undergoing HCRC genetic testing. Furthermore, to the best of our knowledge, no prior study has yet examined the prevalence of the prototypical outcome trajectories following genetic testing. Finally, this is one of the rare studies to have employed the prototypical outcome trajectories to study resilience among an Asian population [27].

## Methods

### Participants and Procedures

Seventy-six consecutive individuals offered genetic testing by the Hereditary Gastrointestinal Cancer Registry (the Registry) in Hong Kong between April 2003 and August 2006 participated in this study after giving informed consent. All of the subjects came from families with proven HCRC syndromes, including familial adenomatous polyposis (FAP) and hereditary non-polyposis colorectal cancer (HNPCC). Among the 76 participants, 71 individuals (43 men and 28 women) from nine FAP and 24 HNPCC families completed the study, including 39 mutation carriers (12 FAP and 27 HNPCC). There were 27 (38.0%) subjects (15 men, 12 women) from FAP families and 44 (62.0%) subjects (28 men, 16 women) from HNPCC families. The mean age was 38.9 years ± 9.2 years (range: 21-66 years). Forty-eight (67.6%) subjects were married , among whom 40 (56.3%) had one or more children. There was no significant difference in personal characteristics between FAP and HNPCC family members.

The participants completed a package of psychological inventories four times during the HCRC genetic testing process: immediately before result disclosure (T1); two weeks after result disclosure (T2); four months after result disclosure (T3); and one year after result disclosure (T4). The first assessment (T1) was conducted at the Registry on the day immediately before result disclosure. A research assistant was available to answer participants' questions. Later assessments (T2 - T4) were conducted at the subjects' homes. Ethical approval was obtained from the Institutional Review Board of Queen Mary Hospital, Hong Kong.

### Measures

Dispositional Hope - The 12-item Adult Trait Hope Scale was rated on the basis of an 8-point Likert scale (1 = definitely false to 8 = definitely true) used to measure hope according to the model of Snyder et al. [17]. A Hope Total score is obtained by aggregating the scores for the 12 items. Because dispositional hope is a trait measure, it was assessed only at T1. The Cronbach's alpha for the total sample at T1 was 0.85.

Anxiety and Depression - The 14-item Chinese version of the Hospital Anxiety and Depression Scale was used to indicate negative emotions [39]. Two scores - HADS Anxiety and HADS Depression- were derived from the questionnaire. Severity of symptom was rated according to a 4-point Likert scale. Higher scores correspond to more symptoms of anxiety and depression, respectively. The 7/8 normative cut-off points for HADS Anxiety and HADS Depression were used to classify participants into low (a score of below or equals to 7) or high (a score of above or equals to 8) anxiety and depression, respectively [39]. For the present sample, the Cronbach's ∝ values for the HADS Anxiety ranged from 0.82 to 0.89 across the four time points (T1 - T4) and those for the HADS Depression from 0.82 to 0.72.

### Data Analysis

Descriptive statistics were provided first and potential syndrome-group (FAP versus HNPCC) and gender differences were examined. Pearson product-moment correlations of the variables at T1 were then analyzed which allowed us to examine the cross-sectional intercorrelational relationships among the psychological variables. The subjects were then classified into different psychological outcome trajectories according to the steps described below (see next section). Finally, logistic regression analyses were used to investigate whether dispositional hope at T1 could predict psychological outcome trajectories after controlling for anxiety or depression level at T1. Given that previous studies show that mutation status (carrier versus non-carrier) is an important factor affecting adjustment [7,8], the interaction of hope and mutation status and the interaction of T1 depression or anxiety and mutation status were also entered into the regression equations.

### Strategies to Establish the Psychological Outcome Trajectories

We used the normative comparison approach to create longitudinal outcome trajectories for psychological functioning using the HADS Anxiety or HADS Depression score at two weeks (T2), four months (T3), and 12 months (T4) post-HCRC genetic testing as separate outcome indicators. The following steps were employed to create the outcome trajectories.

1. We used the 7/8 cut-off of the HADS to classify each subject at each time point as a case (with a score ≥ 8) or a non-case (with a score ≤ 7) [39]. For example, if a subject had a HADS Anxiety score of 9 at T2, then he or she was classified as a HADS Anxiety case at T2. If the same subject had a HADS Anxiety score of 3 at T3, then he/she was considered to be a HADS Anxiety non-case at T3.

2. We mapped all possible combinations of cases and non-cases across the three post-genetic testing result disclosure time points (i.e. two weeks, four months and 12 months post-result disclosure).

3. We categorized participants into one of the four prototypical outcome trajectories [33] using the following operational definitions: *Resilience *was assigned when the subject was a non-case at all three time points; *Chronic Dysfunction *was assigned when the subject was a case at all three time points; *Recovery *was assigned when the subject was a case at T2 but became a non-case at both T3 and T4, or when he or she was a case at both T2 and T3 but became a non-case at T4; *Delayed Dysfunction *was assigned when the subject was a non-case at T2 but became a case at both T3 and T4 or when he or she was a non-case at both T2 and T3 but became a case at T4. Others included all combinations of cases and non-cases across time points other than those described above. Table 1 summarizes the criteria used in defining the four prototypical outcome trajectories in this study.

**Table 1:** Operational Definition of the Four Prototypical Outcome Trajectories

	Two weeks post-result disclosure (T2)	Four months post- result disclosure (T3)	One year post- result disclosure (T4)
Resilience	Non-case	Non-case	Non-case

Chronic	Case	Case	Case

Recovery	Case	Non-case	Non-case
	Case	Case	Non-case

Delay	Non-case	Case	Case
	Non-case	Non-case	Case

## Results

### Intraclass correlation coefficient (ICC)

ICC(1) values of predictors at T1 and psychological indicators at T2 were calculated to examine the independence of the data. All ICC(1) values were < 0.25 (range: 0 to 0.24) and met the criteria for independent measurement[40].

### Psychological characteristics

Due to the unequal group sizes, the powers of the analyses undertaken to detect group differences in syndrome type (FAP versus HNPCC) and mutation status (carrier versus non-carrier) were low to modest, ranging from 0.10 to 0.51. The following results should be interpreted with caution.

Table 2 shows the mean scores of the variables at each time point. HNPCC subjects had a higher level of dispositional hope at T1 than FAP subjects (t(67) = -2.06, p = 0.04). No difference in the anxiety or depression level between the FAP and HNPCC subjects was observed at any time point. Independent sample t-tests were also conducted to examine gender differences. No significant difference for any variable was obtained at any time points. However, subjects with a positive genetic test result had higher HADS Anxiety scores at both T2 and T4. We aggregated HNPCC and FAP subjects for subsequent analyses.

**Table 2:** Descriptive Statistics

	FAP Mean (SD)	HNPCC Mean (SD)	t-value
Time 1 (Immediately before learning genetic result)			

Hope	45.56 (7.05)	49.12 (6.97)	-2.06*
Anxiety	4.26 (3.29)	4.98 (2.96)	-0.95
Depression	3.26 (3.18)	4.23 (2.90)	-1.32

Time 2 (2 weeks)			

Anxiety	3.22 (3.61)	3.95 (3.02)	- 0.92
Depression	2.37 (3.04)	307 (2.57)	- 1.03

Time 3 (four weeks)			

Anxiety	4.00 (3.50)	4.77 (4.12)	- 0.79
Depression	2.67 (3.04)	2.81 (5.69)	- 0.12

Time 4 (one year)			

Anxiety	4.50 (3.37)	5.72 (3.73)	-1.36
Depression	3.44 (2.85)	4.90 (3.53)	-1.80

** p < 0.05			

### Correlation of Variables at Time 1

The correlation coefficients of variables at T1 are depicted in Table 3 to show the cross-sectional relationship of the variables examined in this study. Hope was related to depression, but not to anxiety. Age had no relationship with the three psychological variables investigated in this study.

**Table 3:** Correlates of Psychological Variables among FAP and HNPCC Subjects

	1	2	3	4
1. Hope Total	*	- 0.18	- 0.36**	0.13
2. HADS Anxiety		*	0.65**	0.04
3. HADS Depression			*	0.16
4. Age				*

** p < 0.01

### Psychological Outcome Trajectories

The subjects were classified into different psychological outcome trajectories using the method described previously. The results for HADS Anxiety and HADS Depression are first presented separately.

**Figure 2:**
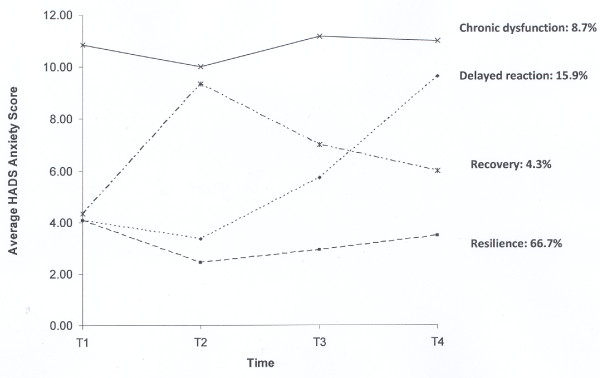
**Outcome Trajectories of HADS Anxiety after HCRC Genetic Testing**. (1) T1 = Immediately before disclosure of genetic testing result; T2 = Two weeks post-result disclosure; T3 = Four months post-result disclosure; T4 = One year post-result disclosure. (2) A small portion of the sample (4.3%) evidenced a variable pattern that could not be categorized into one of the four prototypical patterns was not shown in the figure.

HADS Anxiety as an Outcome Indicator - The trajectories for anxiety are illustrated in Figure 2. The most prevalent was resilience, or stable low anxiety across time. Approximately two-thirds of our subjects exhibited evidence of this trajectory. Delayed reaction, which was characterized by minimal anxiety at T1 and T2 and then a steady increase in the anxiety levels to above the threshold at T4, captured 15.9% of our subjects. The percentages of subjects manifesting a chronic dysfunction trajectory (8.7%) or a recovery trajectory (4.3%) were lower. A small portion of the sample (4.3%) displayed evidence of a variable pattern that could not be categorized into any of the four prototypical patterns.

**Figure 3:**
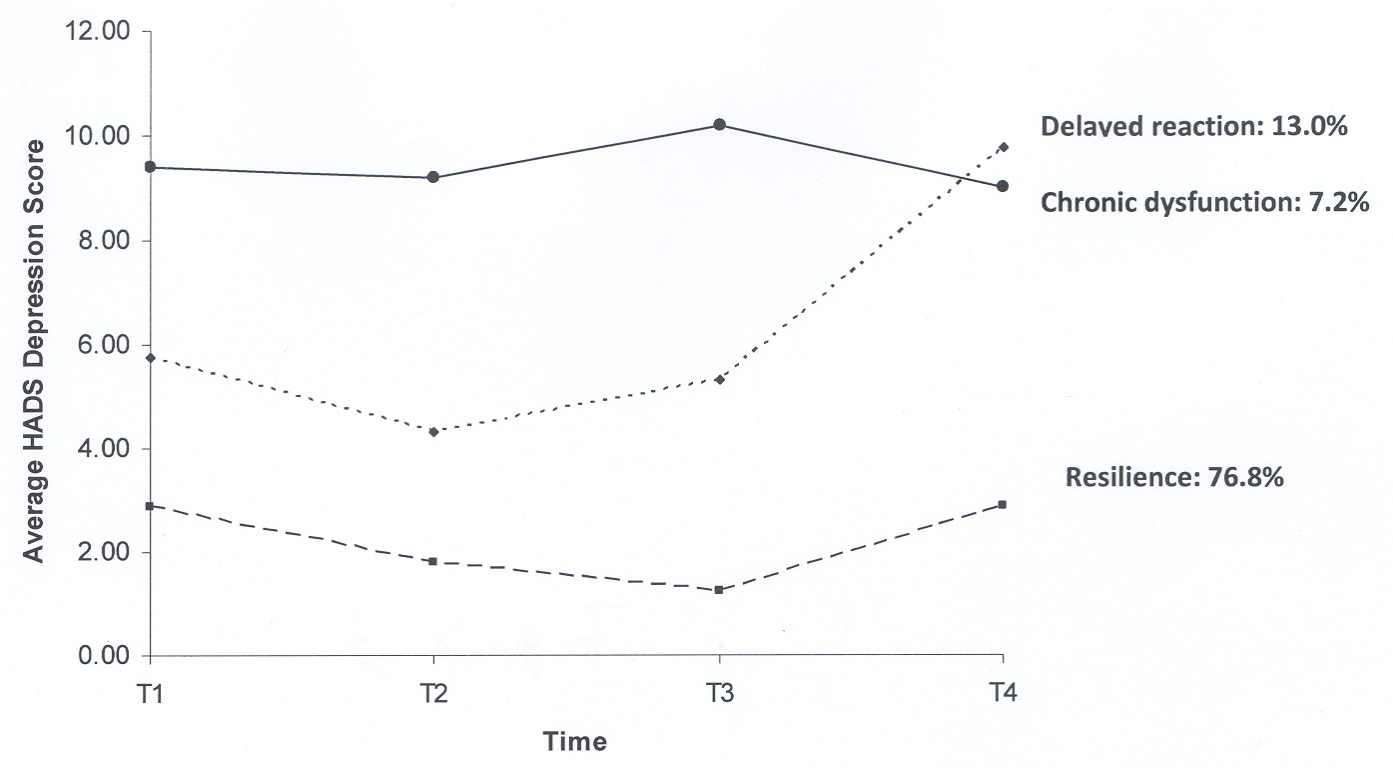
**Outcome trajectories of HADS Depression after HCRC Genetic Testing**. (1)	T1 = Immediately before disclosure of genetic testing result; T2 = Two weeks post-result disclosure; T3 = Four months post-result disclosure; T4 = One year post-result disclosure. (2) A small portion of the sample (2.9%) evidenced a variable pattern that could not be categorized into one of the four prototypical patterns was not shown in the figure.

HADS Depression as an Outcome Indicator - The outcome trajectories for depression are illustrated in Figure 3. Again, resilience (76.8%) was the most prevalent followed by delayed reaction (13.0%). Interestingly, none of the subjects exhibited a recovery trajectory when HADS Depression was used as the outcome indicator. A relatively small percentage of subjects were classified as having chronic dysfunction (7.2%). A small portion of the sample (2.9%) could not be classified in any of the prototypical trajectories.

### Hope to Predict Resilience after HCRC Genetic Testing

Two logistic regressions were then conducted to test whether hope is a reliable predictor of resilience among recipients of HCRC genetic testing. Because very few participants exhibited a recovery trajectory (n = 3 for anxiety and n = 0 for depression), this outcome category was excluded from the regression analyses. The delayed reaction and chronic dysfunction trajectories were obtained into a single non-resilience outcome trajectory because of the small number of subjects in each of these two categories. The dependent variable was therefore a dichotomous outcome trajectory variable: resilience (coded as "1") and non-resilience (coded as "2"). The first logistic regression equation adopted HADs Anxiety as the criterion variable for categorization of the outcome trajectories, whereas the second used HADS Depression.

In both regression equations, the subject's gender (male or female), syndrome group (FAP versus HNPCC), and genetic test result (positive versus negative) were entered in the first step. In step 2, either HADS Anxiety or HADS Depression and the Hope Total score at T1 were entered together with the two interaction terms: Hope x Genetic Testing Results and HADS Anxiety or HADS Depression x Genetic Testing Results. The results of these analyses are presented in Table 4. For both logistic regressions, recipients' dispositional hope at pre-disclosure baseline tended to be a significant individual predictor of resilience (Anxiety: β = -0.11, *p *= 0.05; Depression: β = -0.25, *p *< 0.05). Hence, dispositional hope may predict the resilience of our HCRC genetic testing recipients after controlling for demographic and medical information as well as for mood status prior to test result disclosure.

**Table 4:** Summary of Logistic Regression Analysis for Variables Predicting Resilience versus Other Outcome Trajectories for HADS Anxiety and HADS Depression

	Resilience: HADS Anxiety			Resilience: HADS Depression		
Predictor	***B***	***SE B***	***e***^***B***^	B	***SE B***	***e***^***B***^
Final Step						
Gender	0.33	0.71	1.39	-0.26	0.96	0.78
Syndrome Group	0.93	0.71	1.69	2.48	1.22	11.92
Genetic Testing Results	0.62	5.15	1.85	-8.50	6.91	0.00
HADS Anxiety or HADS Depression at T1	0.22	0.17	1.71	0.56*	0.25	1.72
Hope Total at T1	- 0.11^a^	0.06	0.90	- 0.25*	0.11	0.78
Hope x Genetic Testing Results	-0.03	0.11	0.97	-0.05	0.34	0.95
HADS Anxiety at T1 x Genetic Testing Results	-0.03	0.21	0.97	0.18	0.14	1.12
Constant	1.61	3.01	4.98	3.74	4.89	42.41
*χ*^*2*^	16.53*			35.86**		
*df*	7			7		
Overall Percentage of Correct Classification	74.2%			85.5%		

*Note: e*^*B *^= exponentiated *B*. Gender coded as 0 for *male *and 1 for *female*. Syndrome Group coded as 0 for FAP and 1 for HNPCC. Genetic testing results coded as 0 = positive; 1 = negative; ^a^*p *= 0.05; *p < 0.05; **p < 0.01.

## Discussion

In this study, we used a longitudinal design to assess HCRC genetic testing recipients prospectively on four occasions over a one year period. Our results advance our understanding of the psychological consequences of HCRC genetic testing in several ways. First, the longitudinal design we employed is relatively uncommon among cancer genetic studies and, to the best of our knowledge, the present study is the first to use a prospective design to investigate psychological outcomes of cancer genetic testing among an Asian population. Second, this is the first study to examine the prevalence of different longitudinal outcome trajectories among recipients of HCRC genetic testing. None of the previous studies on HCRC genetic testing has studied outcome trajectories. However, research on these trajectories is becoming an important area of investigation in the field of traumatology [30,33]. Our study provides timely data enabling comparison of the experience of HCRC genetic testing recipients with that of subjects affected by other stressful events. Third, we examined the influence of dispositional hope, a construct that has gained much attention in psychology in recent years, to predict outcome trajectories. This approach is unique among both hope and HCRC genetic testing studies.

The prevalence of outcome trajectories observed in the current study showed that a majority of the HCRC genetic testing recipients were psychologically resilient (66.7% for HADS Anxiety and 76.8% for HADS Depression); that is, these individuals exhibited little or no depression and anxiety from pre-disclosure baseline until one year after disclosure. Our results are consistent with those of other studies showing that predictive genetic testing has no severe psychological impact on its recipients [7,8]. Only a minority of our sample exhibited chronically elevated symptoms (8.7% for HADS Anxiety and 7.2% for HADS Depression). A prior study reported that the percentage of BRCA1/2 or HNPCC genetic susceptibility testing recipients exhibiting an elevated level of anxiety dropped from 29.3% at two weeks to 14.1% at six months after disclosure of the results [6]. Our results suggest that the percentage of recipients showing chronic anxiety and depression levels is likely to drop even further to around 7%-9% at 12 months. This result is consistent with previous observations in Western countries, where typically only 5% to 10% of people exposed to loss or potential trauma tended to experience chronic psychological dysfunction [41], while the majority of individuals observed in these studies tend to exhibit psychological resilience [33]. A recent study on outcome trajectories among Hong Kong patients who had recovered from SARS showed that 35% of the sample displayed resilience, and 42% exhibited chronic dysfunction. In comparison with these SARS survivors, our sample of HCRC genetic testing recipients showed a higher prevalence of resilience and a lower prevalence of chronic dysfunction. Previous studies have suggested that the severity of the stressful event is an important factor affecting the prevalence of outcome trajectories: individuals exposed to extremely stressful events exhibited a higher prevalence of psychopathology and a lower prevalence of resilience in comparison with those who were exposed to low-stress levels [31]. The high prevalence of resilience trajectories and the low prevalence of chronic dysfunction trajectories in our study suggest that HCRC genetic testing induces a milder level of stress in its subjects.

Another finding is that 13%-15% of our subjects exhibited a delayed reaction trajectory, i.e. they reported low levels of anxiety and depression initially but their anxiety and distress levels increased beyond the threshold at T3 or T4. The prevalence of delayed reaction trajectories in this investigation is higher than that observed in previous studies conducted in Western countries (5-10%) but is similar to the 13% reported in a recent study among Hong Kong SARS survivors [30]. It is possible that the perception of less support from the Registry on the anniversary of genetic testing may have caused the elevated anxiety level at T4. Similar elevated psychological distress towards discharge after hospitalization has been reported following other clinical procedures such as bone marrow transplantation [42]. Another possibility is that the HCRC genetic testing results may have a delayed effect on the recipients. For recipients with positive results, the long-term negative consequences of being mutation carriers, such as the inconvenience of regular lifelong medical surveillance and the potential for social discrimination, may only become apparent a few months after result disclosure. For those with negative testing results, adverse psychological reactions such as feelings of guilt for being a non-carrier in the family and communication issues among family members relating to the testing results may only surface some time after result disclosure [13]. However, the supposition that these factors may affect the prevalence of delayed reaction trajectories is tentative and should be investigated in independent studies.

Another objective of the present study was to examine the role of hope [43,44] in affecting the psychological outcome trajectories of HCRC genetic testing recipients. Logistic regression analyses showed that even when the depression and anxiety levels of the subjects at T1 were statistically controlled, hope was still predictive of their resilience trajectories. We believe that high-hope individuals, when confronted with an adverse event such as HCRC genetic testing, are better able to reprioritize their goals in life, better able to generate alternative ideas about how to achieve these goals and have higher levels of motivation to actualize alternative pathways to them[43]. These attributes should be particularly relevant to the adjustment of predictive genetic testing results.

The study has several limitations which should be mentioned. Because our sample size was relatively small, our findings can be generalized only with caution. More importantly, because we had to combine the delayed reaction and chronic dysfunction groups into a single non-resilience group in the logistic regression analysis, we were prevented from investigating the predictive power of disposition hope for each trajectory path. In addition, the unequal group sizes of syndrome type and mutation status also reduced the power of our analyses. Furthermore, other personality measures (e.g. neuroticism) and confounding variables not included in the regression analysis may have influenced the results. For example, we did not measure life events, and it is possible that the participants' reports on their depression and anxiety levels one year after genetic testing have been affected by other factors in their lives. Independent studies with a larger sample size and a more even distribution of group sizes could be carried out in future to examine whether our present findings can be replicated.

## Conclusions

It is possible that high-hope individuals adjust more readily to the results of HCRC genetic testing than their low-hope counterparts. Systematic and empirically-supported hope-based training is now available to increase individuals' hopefulness [45]. In addition to educating subjects about colorectal cancer and genetic testing, hope-based training can be incorporated into genetic counselling programs for individuals undergoing HCRC genetic testing. Systematic outcome studies should be conducted to examine the effectiveness of such hope-based intervention programs which should ideally be provided during the waiting period for genetic testing results.

## Competing interests

The authors declare that they have no competing interests.

## Authors' contributions

SH conceptualized and designed the study. He carried out data analysis; interpretation of the findings as well as wrote the report of this study. JH provided medical advices on the study design and the interpretation of data in this study. She also supervised subject recruitment in this study. GB provided theoretical and statistical advices to the research team. Both AC and EC provided assistance in subject recruitment, data collection and data analysis. All authors read and approved the final manuscript.

## Pre-publication history

The pre-publication history for this paper can be accessed here:

http://www.biomedcentral.com/1471-2407/10/279/prepub
